# An autopsy series of an oft-missed ante-mortem diagnosis: hemophagocytic lymphohistiocytosis

**DOI:** 10.4322/acr.2021.243

**Published:** 2021-04-19

**Authors:** Anusree Majumder, Debraj Sen

**Affiliations:** 1 Armed Forces Medical College and Command Hospital (Southern Command), Department of Pathology and Laboratory Sciences, Pune, India; 2 Armed Forces Medical College and Command Hospital (Southern Command), Department of Radiodiagnosis and Imaging, Pune, India

**Keywords:** Autopsy, Lymphohistiocytosis, Hemophagocytic, Macrophage Activation Syndrome, Ferritins, Hypertriglyceridemia

## Abstract

Hemophagocytic lymphohistiocytosis (HLH) is a rare and potentially fatal syndrome resulting from a hyperactivated immune system. Diverse patient profiles and clinical presentations often result in misdiagnosis. This article describes the varied clinical presentations and autopsy findings in three patients with this entity. The etiopathogenesis of HLH, its disparate and confounding clinical features, the diagnostic criteria, and management principles are also briefly reviewed.

## INTRODUCTION

The immune system normally functions to protect the body from external and internal threats like pathogens and malignancies. At times, however, the immune system can ‘run amok’, and itself be a threat. Hemophagocytic lymphohistiocytosis (HLH) is a potentially life-threatening entity caused by an inappropriate over-activation of the immune system leading to a cytokine storm. Diverse clinical presentations that often overlap with other entities and the lack of sacrosanct diagnostic criteria often result in delayed diagnosis and significant morbidity and mortality.

This article presents the clinical, laboratory, and autopsy findings in three patients with HLH to elucidate the varied and confounding presentations in such patients. The etiopathogenesis, diagnostic criteria, and management principles are also briefly discussed.

## Case 1

A 50-year-old male patient diagnosed with disseminated Tuberculosis, on anti-tubercular therapy, presented with intermittent fever of one-month duration. He also had hepatosplenomegaly. Complete blood counts revealed anemia [hemoglobin (Hb)-8 g/dL (RR for adult males:13-17g/dL) and leukocytosis [total leukocyte count (TLC)-59,000/mm^3^ (RR: 4,000/mm^3^-11,000/mm^3^)] with an abnormal differential leukocyte count (blasts-3%, polymorphs-56%, monocytes-14%, myelocytes-18%, metamyelocytes-06%, lymphocytes-03%). The platelet count was 2 x 10^5^/mm^3^ (RR:1.5 - 4.5 x10^5^/mm^3^). Bone marrow aspiration, along with flow-cytometry, confirmed the diagnosis of Chronic Myelomonocytic Leukemia (CMML), and the patient was started on hydroxyurea therapy. After three months, he developed marked pallor with epistaxis and gingival bleeding. Bone marrow and peripheral smear examination revealed 59% blasts (positive for myeloperoxidase). A diagnosis of Acute Myeloid Leukemia (AML) arising in a background of CMML was made, and he was started on induction chemotherapy. However, he developed severe chemotherapy-induced pancytopenia (Hb-6.5 g/dL, TLC-200/mm^3^, platelet count-2000/mm^3^). Granulocyte-monocyte colony-stimulating factor was started, but the patient developed neutropenic sepsis with coagulopathy and acute renal failure and succumbed to his illness.

## Case 2

**Figure 1:**
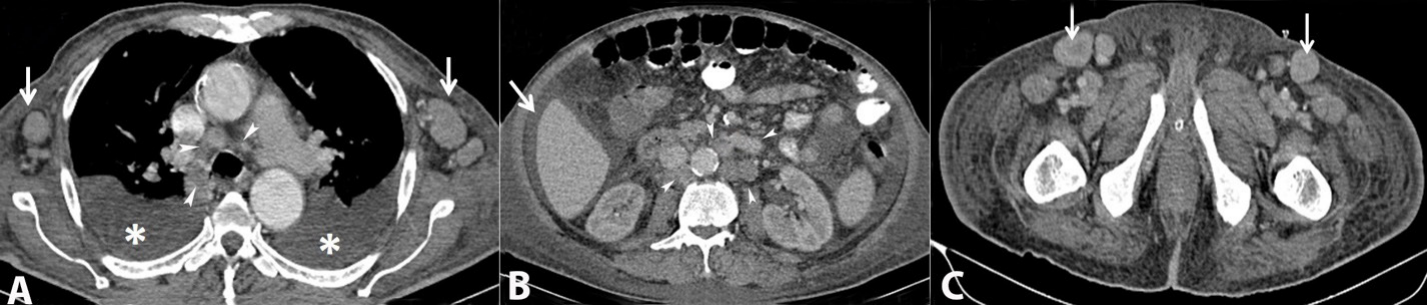
A panel of contrast-enhanced axial CT scan images in soft-tissue window showing: **A** – Thoracic CT - bilateral pleural effusions (asterisk), bilateral axillary lymphadenopathy (arrows) and mediastinal lymphadenopathy (arrowheads); **B** – Abdominal CT - ascites (arrow) and retroperitoneal lymphadenopathy (arrowheads); **C** – Pelvic CT - bilateral inguinal lymphadenopathy (arrows).

A 45-year-old male presented with a high fever, sore throat, cervical lymphadenopathy, and a non-pruritic maculopapular truncal rash of around three weeks duration. He also had polyarthralgia involving small and large joints. History of some unquantified weight loss was also elicited. Axillary and inguinal lymphadenopathy was present. Laboratory investigations revealed normocytic normochromic anemia (Hb-11.2 g/dL), leukocytosis (TLC-26,000/mm^3^) with a neutrophilic predominance, and a normal platelet count. His erythrocyte sedimentation rate (ESR) and C-reactive protein were significantly raised at 120 mm/hr (RR: 0-15mm/hr) and 39 mg/dL (RR: <0.3 mg/dL), respectively. Serology for rheumatoid factor (RA factor), anti-nuclear antibodies (ANA), anti-double-stranded DNA (anti dsDNA) antibodies, anti-neutrophilic cytoplasmic antibodies (ANCA), HLA B-27, and anti-cyclic citrullinated peptide (anti-CCP) antibodies were all negative. He tested negative for Human Immunodeficiency Virus (HIV), Hepatitis B, and Hepatitis C. Venereal Diseases Research Laboratory (VDRL) test was non-reactive. The Mantoux test was negative. Computerized Tomography (CT) of the chest and abdomen revealed mediastinal and retroperitoneal lymphadenopathy with hepatosplenomegaly. Bilateral small pleural effusions and moderate ascites were present (Figure 1).

Cervical lymph node biopsy showed reactive paracortical hyperplasia; no necrosis, granulomata, or atypical cells were seen. Tissue sent for polymerase chain reaction (PCR) assay for Mycobacterium tuberculosis (MTB-PCR) was reported as negative. A diagnosis of adult-onset Still’s disease was considered as per Yamaguchi criteria.^1^ On the fourth day of admission, he developed Acute Respiratory Distress Syndrome (ARDS) with hypotension, severe metabolic acidosis, and acute liver and kidney injury. Laboratory investigations revealed bicytopenia with raised serum lactate dehydrogenase (LDH), triglycerides, ferritin, liver transaminases, and low fibrinogen levels. Peripheral blood smear showed a shift to the left and neutrophilic toxic granulations. ESR showed a drop to 20 mm/hr. In the background of adult-onset Still’s disease, Macrophage Activation Syndrome (MAS) was suspected. Despite aggressive measures, he succumbed to his illness after three days.

## Case 3

**Figure 2:**
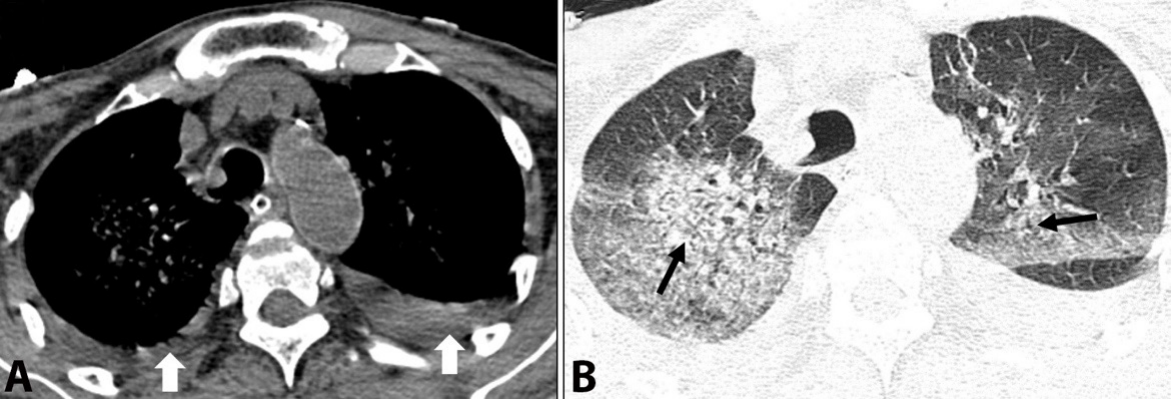
A panel of axial non-contrast thoracic CT scan images showing: **A** – bilateral small pleural effusions (arrows), in the mediastinal window, and **B** – bilateral parenchymal infiltrates in lung window.

A 38-year-old male presented with high-grade fever and sore throat of three days duration. He was initially treated on an out-patient basis with oral antibiotics and antipyretics. After five days, he was brought to the hospital in a state of altered sensorium and severe breathlessness. An urgent CT chest was performed that showed bilateral parenchymal infiltrates associated with pleural effusions (Figure 2). He had two episodes of generalized tonic-clonic seizures followed by cardiac arrest soon after arrival at the hospital, following which he was put on mechanical ventilation.

**Figure 3:**
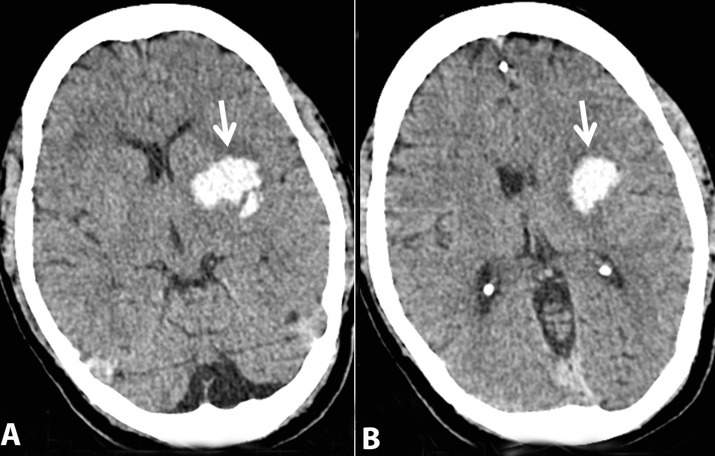
Axial non-contrast CT scan images of the brain showing a hematoma in the left gangliocapsular region (arrows).

Peripheral blood smear showed pancytopenia with features of hemolysis, relative lymphocytosis, and activated lymphocytes. His liver and renal function tests and coagulation profile were deranged. An urgent CT scan of the head revealed hemorrhage in the left gangliocapsular region (Figure 3).

He suffered a second cardiac arrest within 24 hours of hospital admission and could not be revived. The cause of death was attributed to MODS (multi-organ dysfunction syndrome), secondary to an infective febrile illness of possible viral etiology. The salient laboratory findings of the three patients are summarized in Table 1.

**Table 1:** Ante-mortem laboratory parameters of patients

	Case 1	Case 2	Case 3	Normal range
Diagnosis	CMML in blast crisis	Adult-onset Still’s disease	Septic shock, acute respiratory illness (? Viral)	
Hb (g/dl)	6.5	10.2	6.8	12-17 g/dl
TLC (/mm^3^)	200	26,000	1,900	4000-11,000
DLC	PMN 20%, Ly 80%	PMN-90%(Bands-30%), Ly-10%	PMN-32%, Ly-60%, Mo-08%	
Platelets (/mm^3^)	2,000	8,0000	10,000	1.5-4.5 x 10^5^
INR	3.25	3.75	5.25	
ESR (mm/hr)	46	120	68	<20
CRP (mg/L)	160	89	78	<0.3
bilirubin (mg/dL)	2.3	1.8	3.4	0.2-1.2
AST (IU/L)	389	470	850	5-40
ALT (IU/L)	288	397	679	7-56
Ferritin (mcg/L)	6900	18500	11980	20-250
Triglycerides (mg/dL)	270	494	342	<150
LDH (IU/L)	895	4482	3376	140-280
Fibrinogen (g/L)	-	1.8	1.0	2-4

ALT= alanine aminotransferase, AST= aspartate aminotransferase, CMML=chronic myelomonocytic Leukemia, DLC= differential leukocyte count, ESR= Equipment sedimentation ratio, Hb= hemoglobin, INR= International normalized ratio, LDH= lactate dehydrogenase, Ly= lymphocyte, M= monocyte, PMN= polymorphonuclear, TLC= total leukocyte count. In case 3 - presence of schistocytes, polychromasia and nucleated RBC in the peripheral blood smear.

## AUTOPSY FINDINGS

Autopsies were performed to ascertain the cause of death in all three patients. All viscera were preserved for histopathological examination.

### Salient gross findings

#### Case 1

**Figure 4:**
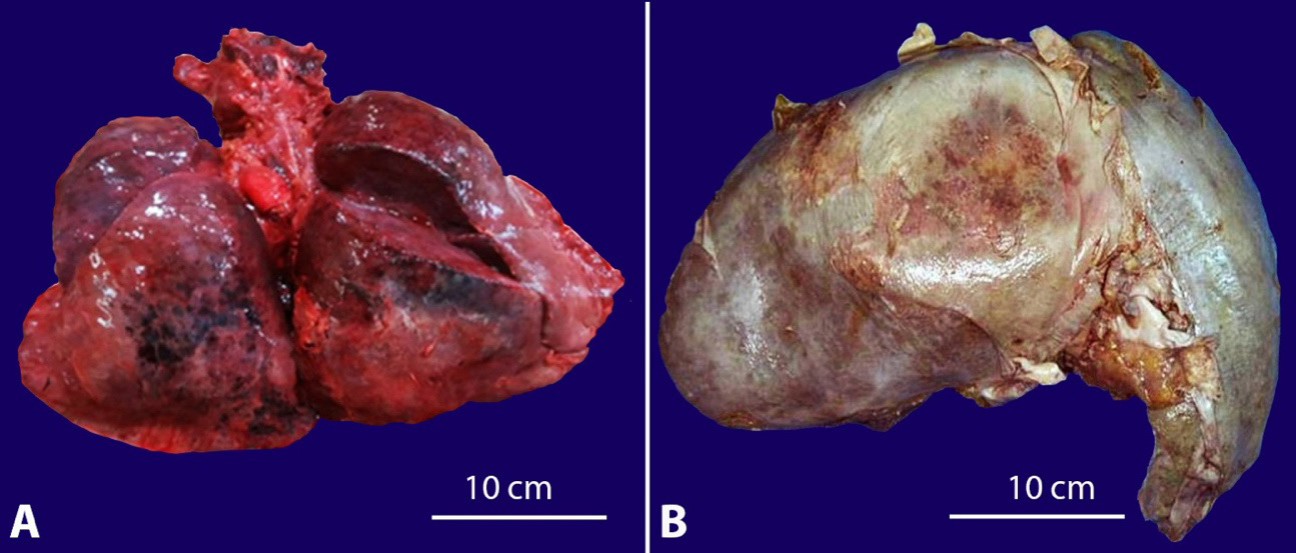
Gross view of organ specimens from case 1 showing: **A** – heavy, congested lungs, and **B** – an enlarged liver with rounded anterior margins.

The deceased was cachectic with the presence of pallor and bilateral pedal edema. Multiple petechial spots were seen over the thorax and abdomen. The lungs were heavy and congested (combined weight-1250g; RR:685g-1050g) (Figure 4A). Bilateral serosanguineous pleural effusions were noted (200 ml). The spleen was enlarged and congested (weight-175g; mRR-155g). The liver was enlarged (weight-1890g; RR: 1500 g-1800g), and the anterior margin was rounded; the cut surface was unremarkable (Figure 4B). Multiple paratracheal, paraaortic, and peripancreatic lymph nodes were seen. 500ml of serosanguineous ascitic fluid was noted. Other organs were grossly unremarkable. Postmortem cultures of heart blood, pleural, peritoneal, and cerebrospinal fluid grew *Klebsiella spp.*

#### Case 2

**Figure 5:**
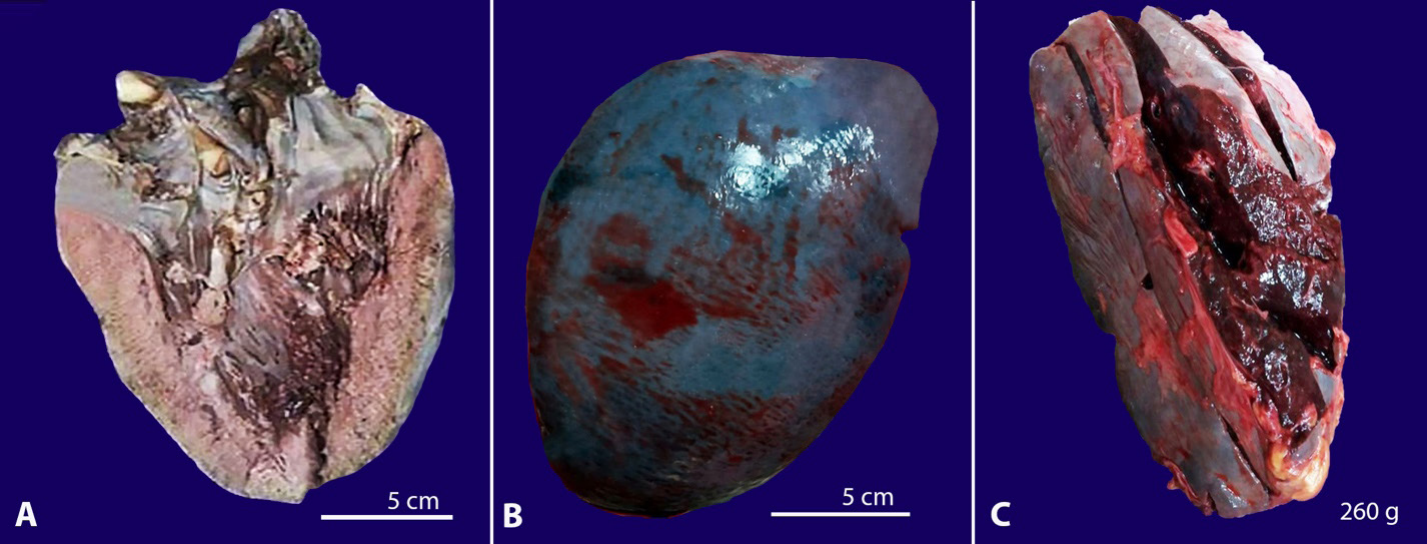
Photographs of organ specimens from case 2: **A** – cut section of heart showing thick ventricular walls, **B** – gross view of the enlarged congested spleen, and **C** – cut section of the spleen.

The subject was averagely built and nourished. A maculopapular truncal rash with multiple petechial spots over the lower limbs was noted. Both lungs were subcrepitant and edematous with red, hyperemic cut surfaces (combined weight-1245g). Bilateral serosanguineous pleural effusions (500 ml) were noted. Multiple enlarged hilar and mediastinal lymph nodes were seen. The heart weighed 430g (RR: 270-360g). The right and left ventricular walls were thick and measured 1.5 cm and 2 cm in thickness, respectively (mRR: right ventricle-0.5cm, left ventricle-1.5cm) (Figure 5A). No mural thrombus/ infarct was seen. The liver was enlarged (weight-1750 g); the cut surface was unremarkable. The spleen was enlarged (weight – 260g; mRR-155g) and congested (Figures 55C). Abdominal lymphadenopathy was noted. Cultures of heart blood and pleural fluid showed no growth.

#### Case 3

The subject was averagely built and nourished. The lungs were congested (combined weight-1420g). Bilateral sero-sanguineous pleural effusions (500 ml) were present.

**Figure 6:**
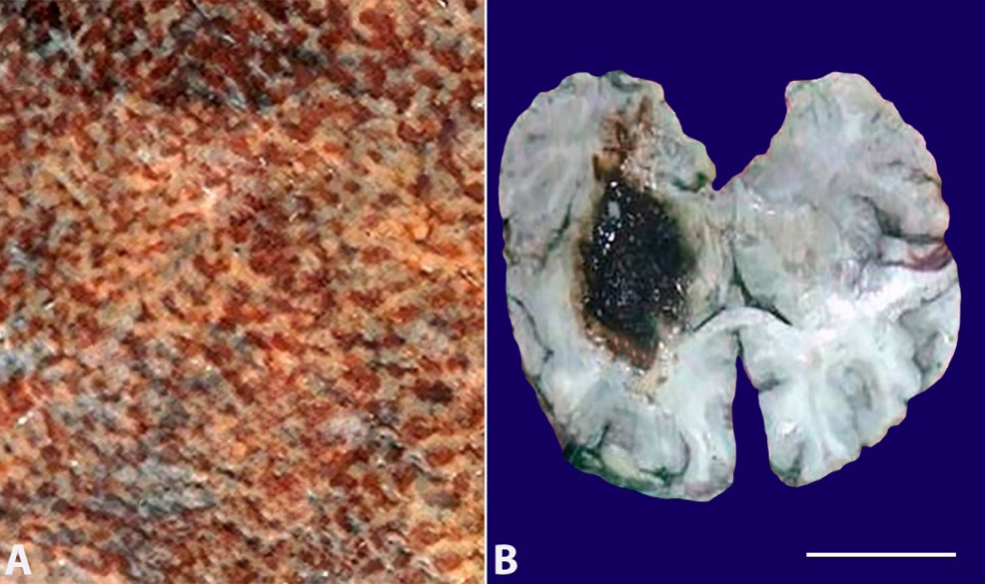
**A** – Detail of a cut sections of the liver with a variegated and mottled appearance of the liver (nutmeg liver), **B** – a hematoma in left basal ganglia (scale bar = 7 cm).

Multiple paratracheal and peribronchial lymph nodes were noted. The liver was enlarged, tense, and cyanotic (weight-1960g); the cut surface appeared variegated and mottled red (Figure 6A). The spleen was enlarged, congested, and friable (weight-180 g). The brain weighed 1450g (RR:1100-1700g). On the cut section, a hematoma measuring 3.5 cm x2.5cm was seen in the left basal ganglia extending into the left ventricle (Figure 6B). No evidence of cerebral herniation was noted. Post-mortem tracheal and nasopharyngeal aspirates were tested for Influenza A & B, pandemic A (H1N1), Respiratory syncytial viruses (RSV A & B), Human Metapneumovirus (hMPV), Parainfluenza viruses (PIV 1-4), Rhinovirus, Adenovirus. The aspirate was positive for (H1N1). Cultures of heart blood and pleural fluid showed no growth.

### Salient histopathological findings

**Figure 7:**
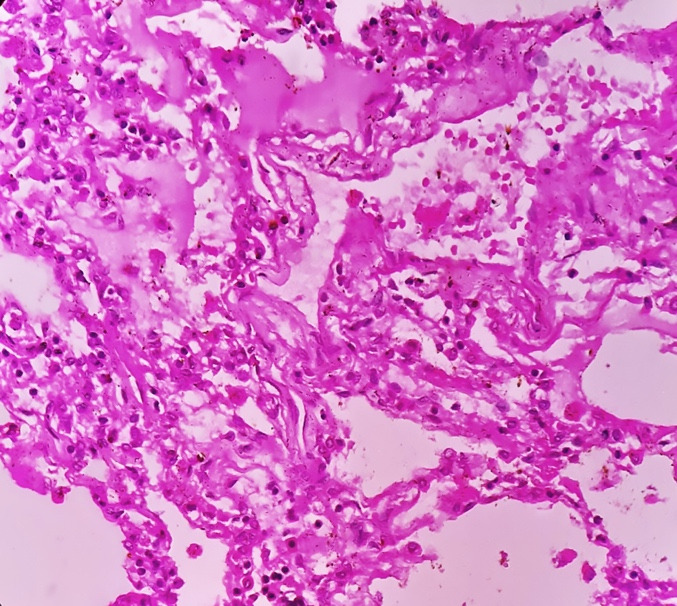
A photomicrograph of lung showing interstitial and intra-alveolar accumulation of mixed inflammatory infiltrate and waxy, hyaline membrane (H&E, 40X).

In all three cases, sections from the lungs showed features of diffuse alveolar damage. There was congestion along with an interstitial and intra-alveolar accumulation of fluid, fibrin, and the presence of mixed inflammatory infiltrate. The alveolar walls were lined with waxy, hyaline membranes. A variable number of hemosiderin-laden alveolar macrophages (heart failure cells) was noted (Figure 7).

**Figure 8:**
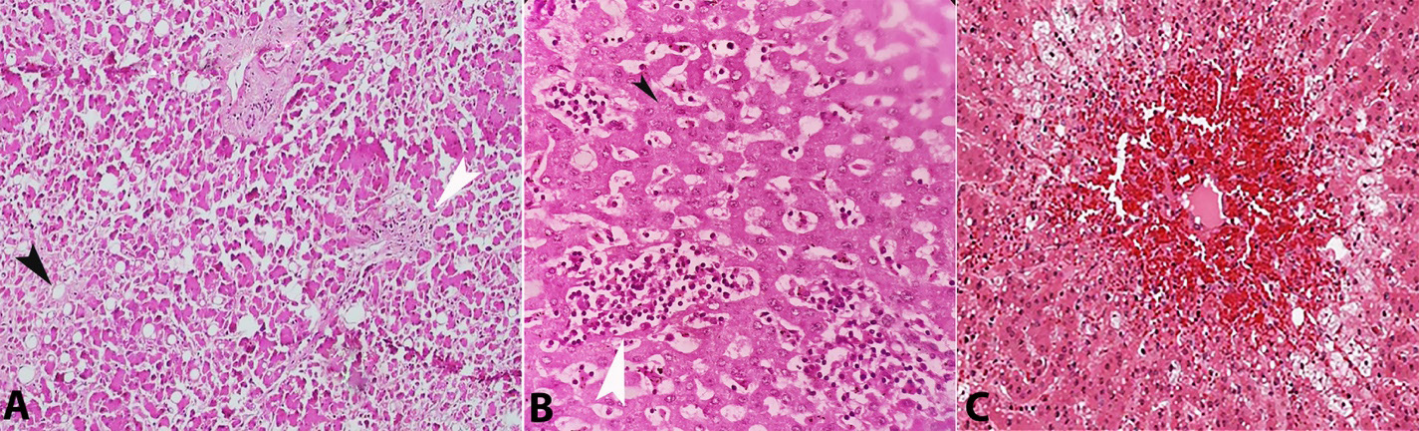
Photomicrographs of the liver showing: **A** – steatosis (black arrowhead) with periportal inflammatory infiltrate (white arrowhead) in case 1 (H&E 10X); **B** – dilated hepatic sinusoids (white arrowhead) with spotty necrosis (black arrowhead) in case 2 (H&E, 40X magnification); and **C** – centrilobular congestion and hemorrhage in case 3 (H&E, 10X magnification).

Sections from the liver in cases 1 and 2 showed chronic hepatitis-like features (steatosis, spotty necrosis, sinusoidal congestion, and dilatation with periportal lymphomononuclear inflammatory cell infiltrate). In case 3, there was centrilobular congestion, hemorrhage, and necrosis (Figure 8).

**Figure 9:**
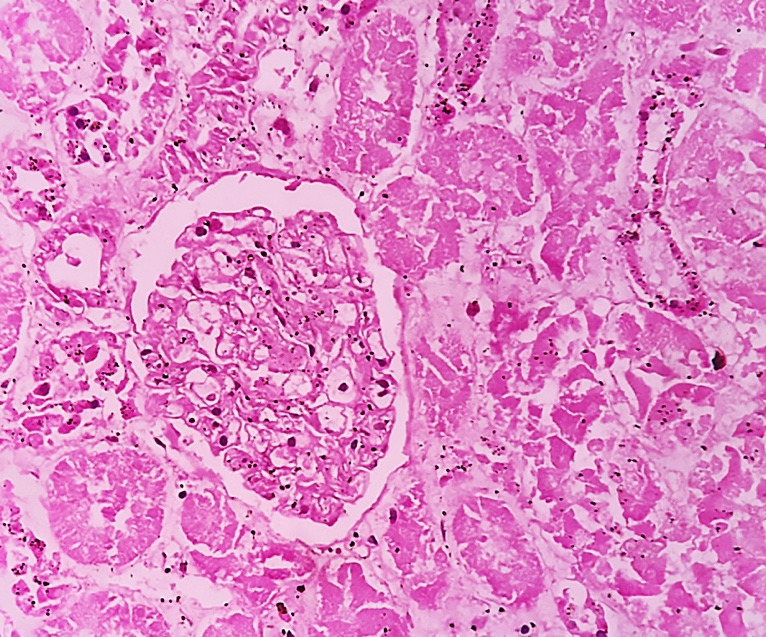
A photomicrograph of kidneys showing extensive tubular necrosis (H&E, 40X).

Sections from the kidneys in all cases showed features of acute tubular necrosis with desquamation of epithelial cells into the tubular lumen (Figure 9).

**Figure 10:**
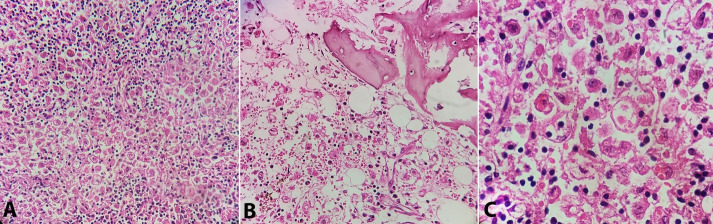
Photomicrographs showing hemophagocytosis by macrophages in **A** – lymph node (H&E, 10X magnification), **B** – bone-marrow (H&E, 10X magnification), and **C** – spleen (H&E, 40X magnification).

Diagnostic histopathological findings of HLH were noted in the lymph nodes, bone marrow, and spleen in all three cases. Massive sinusoidal dilatation and infiltration by histiocytes engorged with phagocytosed erythrocytes, granulocytes, and lymphocytes were seen in lymph node sections (Figure 10A). Smears from bone-marrow showed depleted hematopoietic precursors along with the proliferation of macrophages exhibiting hemophagocytosis (Figure 10B). Splenic sections revealed attenuated white pulp with extensive infiltration by hemophagocytic histiocytes (Figure 10C).

**Figure 11:**
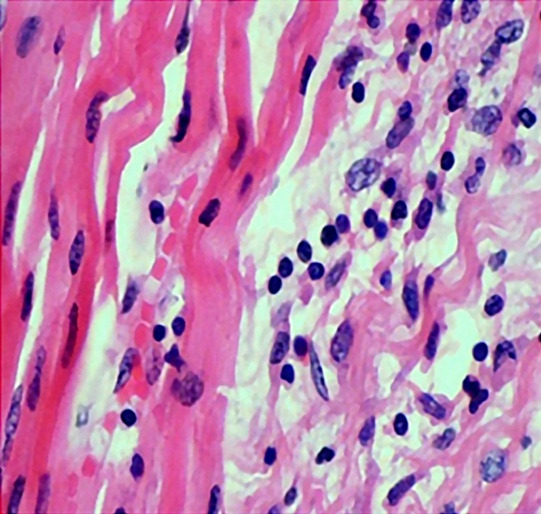
A photomicrograph showing lymphocytic infiltrates in the myocardium (H&E, 40X magnification).

Lymphocytic myocarditis was noted in the posterior wall of the left ventricle in case 2 (Figure 11). Immunohistochemistry (IHC) panel comprising CD3, CD68, CD45RO was employed to demonstrate myocarditis (as per Dallas criteria guidelines). This finding can be attributed to the patient’s underlying autoimmune disease.

**Figure 12:**
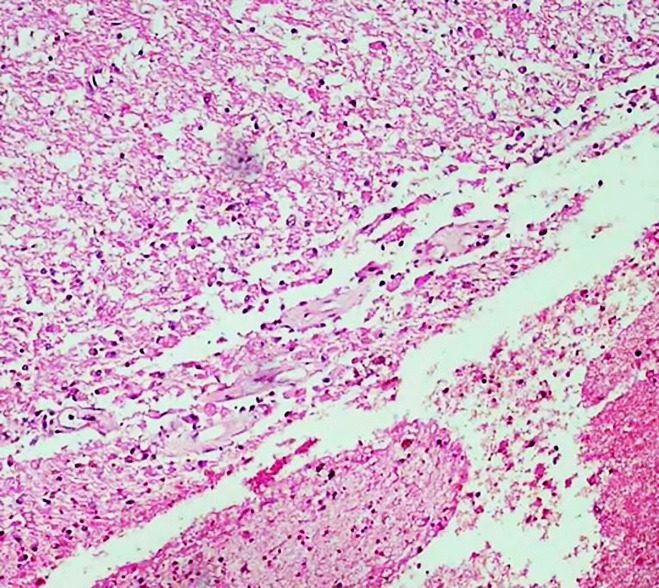
A photomicrograph showing liquefactive necrosis of brain parenchyma with hemorrhage (H&E, 10X magnification).

In case 3, sections from the brain showed left gangliocapsular hematoma (and liquefactive necrosis) (Figure 12). Cerebral edema was suggested by the prominence of Virchow Robin’s spaces, swelling of neurons with a widening of perivascular spaces, and congestion of blood vessels. No granuloma/ organism was seen. In cases 1 and 2, sections from the brain were largely unremarkable except for some mild cerebral edema in case 2.

The autopsy findings are tabulated in Table 2.

**Table 2:** Autopsy findings in all three patients with HLH

	Case 1	Case 2	Case 3
Gross findings	Heavy, boggy lungs Ascites; sero-sanguineous pleural effusion; hepatosplenomegaly; multiple paratracheal; para-aortic peripancreatic lymph nodes.	Heavy, boggy lungs; Sero-sanguineous pleural effusion; cardiomegaly; multiple thoracic, abdominal lymph nodes; hepatosplenomegaly.	Heavy, boggy lungs; sero-sanguineous pleural effusion; nutmeg liver; multiple enlarged paratracheal; peribronchial lymph nodes; congestive splenomegaly; left gangliocapsular hematoma.
Heart blood/CSF/ Ascitic/ Pleural culture	*Klebsiella pneumonia*	No growth	No growth
Post-mortem tracheal & nasopharyngeal aspirate	-	-	Pandemic A (H1N1)
Microscopy	Hemophagocytosis in bone-marrow, spleen & lymph nodes; diffuse alveolar damage lungs; steatosis with spotty necrosis & periportal inflammation in liver, acute tubular necrosis in kidneys	Hemophagocytosis in bone marrow, spleen & lymph nodes; lymphocytic myocarditis**;** diffuse alveolar damage lungs**;** steatosis with periportal inflammation & spotty liver necrosis**;** acute tubular necrosis in kidneys	Hemophagocytosis in bone marrow, spleen & lymph nodes; diffuse alveolar damage lungs; acute passive congestion in liver, acute tubular necrosis in kidneys; left gangliocapsular hematoma with liquefactive necrosis of brain parenchyma

‘CSF’ stands for ‘Cerebrospinal fluid’.

## DISCUSSION

HLH is a rare, life-threatening disorder characterized by an inappropriate hyper-activation of the immune system. Farquhar and Claireux first described it in 1952 in siblings.^2^ ‘Primary’ or ‘Familial’ HLH is a rare entity occurring in infants and young children that results from mutations in genes regulating the function of the cytotoxic T-lymphocytes (CTLs) and the natural killer (NK) cells.^3^ In contrast, secondary or acquired HLH may be triggered by infections, malignancy, or autoimmune diseases.^2^ ^-^ ^5^ The annual incidence is reported to be approximately 1.2 cases per million, though it is likely that many cases go undiagnosed.^6^ Macrophage Activation Syndrome (MAS) is a subset of HLH which occurs in the setting of autoimmune diseases like systemic-onset juvenile idiopathic arthritis (sJIA), and its adult equivalent adult-onset Still’s disease, systemic lupus erythematosus, Kawasaki disease, and some periodic fever syndromes.^7^

Based on data accrued from HLH-94 (the first prospective international treatment protocol sponsored by the Histiocyte Society) as well as other studies, the Histiocyte Society treatment protocol HLH-2004 was formulated which laid down the diagnostic guidelines for HLH (Table 3).^9^

**Table 3:** Diagnostic Criteria for HLH/MAS

Based on HLH 2004			2016 classification criteria for MAS complicating sJIA (Ravelli et al*.* ^8^)
1. Molecular diagnosis (Confirmed HLH associated genetic mutation)			A known or suspected patient of sJIA with fever is classified as having MAS if the following criteria are met:
Or			· Ferritin >684ng/ml and
2. Fulfilment of 5 out of the following 8 criteria:			· Two of the following:
(a) Fever			o Platelet count ≤ 181x10^9^/l
(b) Splenomegaly			o AST> 48U/l
(c) Cytopenias in peripheral blood (affecting at least 2 hematopoietic cell lineages):			o Triglycerides >156 mg/dl
- Hemoglobin < 90 gm/L			o Fibrinogen≤360mg/dl
(<100gm/L in infants < 4 weeks of age)			
-Platelets < 10x10^9^ /L			
-Neutrophils <10x10^9^/L			
(d) Hypertriglyceridemia and/or hypofibrinogenemia:			
-Fasting triglycerides ≥3.0 mmol/L (ie, ≥265 mg/dL)			
-Fibrinogen ≤1.5 g/L			
e) Hemophagocytosis in the bone marrow, spleen, or lymph nodes			
f) Low or absent natural killer cell activity			
g) Ferritin ≥500 ng/mL			
h) Soluble CD25 (ie, soluble interleukin-2 receptor) ≥2,400 U/mL			
**Based on the ‘H’ Score**			
**Parameter**	**N^o^ of points**	**H score**	**Probability of Hemophagocytic syndrome**
Known underlying immunosuppression	0 (no) or 18 (yes)	90	<1
Temperature (^0^C)	0(<38.4),	100	1
	33 (38.4-39.4) 49(>39)	110	3
Organomegaly	0(No)	120	5
	23(Hepatomegaly or splenomegaly)	130	9
	38(Hepatomegaly and splenomegaly	140	16
No of cytopenias	0(1 lineage)	150	25
	24(2 lineages)	160	40
	34 (3 lineages)	170	54
Ferritin (ng/ml)	0(<2000)	180	70
	35(2000-6000)	190	80
	50(>6000)	200	88
Triglyceride (mmol/L)	0(<1.5)	210	93
	44(1.5-4)	220	96
	64(>4)	230	98
Fibrinogen (g/L)	0(>2.5)	240	99
	30(≤2.5)	250	>99
Serum glutamic oxaloacetic transaminase (IU/L)	0 (<30)		
	19(≥30)		
Hemophagocytosis features on bone marrow aspirate	0 (No)		
	35(Yes)		
Hb ≤ 9.2gm/dl; Leukocyte count ≤5000/mm^3^; Platelet count ≤1,10,000/mm^3^			
HIV positive/On long term immunosuppressive therapy (Glucocorticoids/Cyclosporine/Azathioprine)			
Best cut-off for H score - 169			

However, it includes criteria like the assessment of Natural Killer (NK) cell activity and soluble CD 25 levels, facilities for which are not widely available. Hence, the *H score* formulated by Fardet et al.^10^ may be a more practical diagnostic alternative (Table 3).

In our series, all three patients presented with high fever, hepatosplenomegaly, lymphadenopathy, and MODS features (acute respiratory distress syndrome, acute kidney, and liver injury with coagulopathy). Pancytopenia was documented in two patients, while one patient presented with neutrophilic leukocytosis and a normal platelet count. Serum ferritin, triglycerides, and LDH were significantly elevated in all. In all cases, hemophagocytosis was demonstrated in the bone marrow, spleen, and lymph nodes at post-mortem. The calculated H-score with probability for HLH in our patients is depicted in Table 4.

**Table 4:** H-score of the three patients

	H score	Probability of HLH
Case 1	287	>99
Case 2	327	>99
Case 3	299	>99

While two patients had severe leukopenia, one had leukocytosis. This finding has been documented in cases of MAS.^7^ ^,^ ^11^ ^,^ ^12^ MAS is considered a diagnostic challenge as symptoms are non-specific, appear late, and are difficult to distinguish from a concurrent flare-up of the underlying autoimmune condition or coexisting systemic infection. Moreover, patients with autoimmune diseases often have preexisting leukocytosis, thrombocytosis, hyperferritinemia, and hyperfibrinogenemia as a part of the underlying inflammatory condition. In such patients, even with the onset of MAS, the TLC/platelet count/ fibrinogen level may be paradoxically normal. Hence, the cut-off values for cytopenias, ferritin, and fibrinogen levels, as defined in HLH-2004, may not be applicable. Based on expert opinion, available medical literature, and real patient data, Ravelli et al.^8^ proposed diagnostic criteria for MAS in patients of sJIA. These diagnostic criteria include 5 laboratory variables, along with one clinical criterion (fever) (Table 3). In case 2 of our series, 4 out of these 5 variables (ferritin, platelet count, AST, triglycerides) showed a >50% rise from pre-MAS values, out of which serum ferritin showed the most significant rise. Minoia et al.^13^ and Kostik et al.^14^ also tried to define the best cut-off points for laboratory parameters and the most relevant clinical symptoms for diagnosing MAS. However, all these studies were conducted in pediatric patients of sJIA, and their applicability in adult patients is debatable. The clinical picture and laboratory parameters may get further vitiated in the setting of active treatment with IL-1 or IL-6 blockade.^7^

In case 2, there was a notable change in the pattern of fever post-MAS. From intermittent high grade in nature, the fever pattern became continuous non-remitting with the onset of MAS. The laboratory parameters also consistently pointed to a MAS diagnosis, while the serum ferritin rose to 18,500mcg/L (from 5780 mcg/L), his TLC, platelet count, and fibrinogen also showed a >50% rise from baseline levels. This suggests that daily laboratory parameters monitoring to detect subtle alterations may be crucial in the early diagnosis of MAS. A rise in ferritin coupled with a concomitant fall in ESR (due to consumption of fibrinogen and decreased hepatic synthesis) may serve as an early marker of MAS. Ferritin to ESR ratio of ≥ 21.5 was found to have 82% sensitivity and 78% specificity in distinguishing MAS from an autoimmune flare.^15^

At autopsy, the lungs showed features of diffuse alveolar damage with serosanguineous pleural effusion in all cases; this finding was likely to be a part of the spectrum of MODS. Limited literature is available on lung involvement in HLH/MAS. Seguin et al.^16^ found lung involvement in 118 (54%) out of 219 patients with HLH. Dyspnea and cough were the most common symptoms. On imaging, alveolar and interstitial infiltrate, centrilobular nodules, ground-glass opacities, consolidation, pleural effusion, and mediastinal lymphadenopathy were noted. Underlying causes of lung involvement included infections in 52 patients, pulmonary edema associated with heart failure in 34 patients, and malignancies (lymphoma) in 22 patients. Lung involvement in HLH carried a poor prognosis with a mortality rate of 52.5% versus 20% in patients without pulmonary involvement.^16^ A few studies have also documented pulmonary hemorrhage, bronchiolitis, and lymphangitic spread of mononuclear cells.^17^ ^,^ ^18^ Hemophagocytosis has been reported in the lungs in pediatric patients.^19^

Liver findings in HLH have been well studied.^20^ ^,^ ^21^ Hyperbilirubinemia with elevated transaminases [Aspartate transaminase (AST)>Alanine aminotransferase (ALT)] reflects the degree of liver dysfunction. Four distinct histological patterns have been described: (a) chronic hepatitis-like, (b) leukemia-like, (c) histiocyte storage disorder-like, and (4) neonatal giant cell hepatitis-like.^21^ The most common pattern is a chronic hepatitis-like picture characterized by Kupffer cell hyperplasia, portal, and sinusoidal infiltrate of cytotoxic T-cells with a variable number of hemophagocytic histiocytes. Varying degrees of the portal and central vein endothelialitis, lymphocytic bile duct injury, sinusoidal congestion, and dilatation along with non-specific features like hepatocellular necrosis, steatosis, cholestasis, and hemosiderosis are seen.^21^ In our autopsy series, liver histology demonstrated a chronic hepatitis-like picture in two cases, while one case had non-specific centrilobular hemorrhagic necrosis, probably secondary to circulatory failure. However, no hemophagocytosis could be demonstrated (Table 2). Lymphocytic myocarditis was demonstrated in one case (Case 2).

Hemophagocytosis, which is the histopathological *sine qua non* finding for the diagnosis of HLH, has been demonstrated in the bone marrow, spleen, lymph nodes, lungs, liver, skin, meninges, CSF, and rarely in subcutaneous tissue.^4^ ^-^ ^6^ ^,^ ^22^ ^-^ ^24^ However, hemophagocytosis is often cyclical, which may necessitate multiple bone marrow biopsies.^22^ ^-^ ^24^ Moreover, hemophagocytosis is not specific to HLH; it has also been noted in 60% of patients with severe sepsis who do not fulfill the criteria for HLH.^24^ This has been attributed to the suppression of NK cell activity in severe sepsis. These findings point to a need to review the HLH–2004 guidelines, which include hemophagocytosis as one of the defining criteria for HLH.

A notable finding in HLH is the central nervous system (CNS) involvement. More commonly seen in pediatric patients with familial HLH, cerebral HLH is rare in adults and, when present, is usually associated with a high degree of mortality and neurological sequelae. In a study by Cai et al.^25^, of 289 adult patients with HLH, only 10% had CNS involvement. The symptoms range from seizures, altered mental state, meningitis, ataxia, hemiplegia, encephalopathy, cranial nerve palsies to simple irritability. Magnetic Resonance Imaging (MRI) may reveal nodular parenchymal lesions along with leptomeningeal enhancement, demyelination, atrophy, and acute necrotizing encephalopathy (ANE). Isolated cases of subdural hemorrhage have also been reported.^26^ ^,^ ^27^ An uncommon but fatal finding is the intracerebral hemorrhage, which may be a consequence of DIC that commonly accompanies HLH. Cerebrospinal fluid (CSF) analysis in suspected cases of cerebral HLH may reveal pleocytosis, increased proteins, and occasionally hemophagocytosis.^28^ However, most cases have non-specific neuroimaging and CSF findings. In our study, one patient presented with an altered mental state and was detected to have intracerebral hemorrhage on CT scan. This suggests the need for early imaging in HLH patients with CNS symptoms.

Mutation analysis studies should ideally be performed in all suspected HLH patients as characteristic gene mutations preclude the need to fulfill other diagnostic criteria. Tests include measurements of NK-cell activity by the 51-Cr release assay and soluble CD25 (sCD25) levels by immunoassay. Emerging biomarkers like soluble CD163, IL-18, and FSTL-1 (follistatin-related protein 1) may be considered in the diagnosis of MAS.^29^ ^,^ ^30^ However, their availability and application remain a challenge.

The HLH 94 treatment protocol consists of corticosteroids, cyclosporine A, intrathecal therapy with Methotrexate, and etoposide. However, the heterogeneity of HLH calls for individualized treatment protocols, particularly in elderly patients with comorbidities who are more prone to end-organ damage. In acquired HLH, identification and concurrent treatment of the underlying cause is important. Cases of refractory HLH may benefit from a combination of intensified chemotherapy and allogeneic stem cell transplant. Some of the salvage agents approved in refractory cases include alemtuzumab (anti-CD 52 antibody) and emapalumab (anti-IFNy monoclonal antibody). A notable addition to the arsenal is tocilizumab (anti-IL-6 antibody), which has been used to treat cancer patients with immunotherapy induced cytokine storms.^31^

## CONCLUSION

This article highlights the varied clinical and biochemical profile and autopsy findings in three patients with HLH. Cytopenias that are widely regarded as definite diagnostic criteria for HLH may present late or even be absent. Hemophagocytosis may be cyclical and is not specific to HLH. Also, a diagnosis of HLH may be confounded by preexisting hepatosplenomegaly in patients with underlying hematolymphoid malignancies, infections, and auto-immune disorders. Thus, deranged liver and renal function, coagulopathy along with elevated ferritin and triglycerides may be early harbingers of this condition. Though our understanding of the complex pathophysiology of HLH has increased, much work remains to be done for unambiguous early diagnosis and management of this oft-missed and potentially fatal entity.
